# Advice to a Mother on the Management of Her Offspring

**Published:** 1862-07

**Authors:** 


ADVICE TO A MOTHER ON THE MANAGEMENT OF HER OFFSPRING. By PYE HENRY CHEVASSE, M.D. Fellow of the Royal College of Surgeons of England; Author of "Advice to a Wife on the Management of her own Health," etc. Reprinted from the sixth edition, by BAILLIERE BROTHERS, 440 Broadway, New York, 1862.

This is a small volume of 175 pages, very properly addressed to mothers. It is arranged in the form of questions and answers, and the most perfect simplicity of style is preserved throughout.

It embraces almost every topic, that can arise in the physical and mental management of children from birth to puberty. The directions of the author are so plain and judicious, that we commend the work strongly to the attention of parents and all others having the care of children. It might, also, be usefully consulted by many practitioners of medicine.

